# Pivot Teeth

**Published:** 1868-04

**Authors:** 


### ARTICLE IV.

### *Pivot Teeth.*

By the following method, which we obtained in a conversation with Dr. T. J. Thomas, a member of the late Graduating Class of the Baltimore College of Dental Surgery, artificial crowns can be attached to natural roots, and what in other cases is the exposed portion of the root, perfectly protected from the action of deleterious agents. Prepare the root, as for an ordinary wooden point ; then select a plate tooth of the proper size, shape and shade, and fit it by grinding accurately to the prepared root.

After this is done enlarge the pulp canal by reaming it out as large as the root will admit: that is, make a conical shaped cavity in the exposed surface of the root, allowing the margin of this cavity to be quite near to the circumference of the root, with slight undercuts on the anterior and posterior walls.

After this cavity is prepared, and that portion of the pulp canal beyond it, filled to the apex of the root with gold, make a square metallic pivot of twenty carat gold alloyed with platinum in the proportion of five parts of gold to one of platinum. This pivot is made in two parts, which parts are soldered together at the base of the artificial crown, and slightly wedge shaped.

After this is prepared a thin piece of platinum plate is bent around the pivot, thus making a square cylinder into which the pivot perfectly fits. After this is done, carefully draw the pivot out of the square cylinder and solder the edges of the cylinder with pure gold. The pivot is then returned to the cylinder, and the excess of solder and also any rough edges which may exist on the cylinder filed off. After this is done the cavity in the root is carefully dried of all moisture and protected from saliva by means of napkins, and the square tube or cylinder, with the pivot inside of it, is placed in the centre of this cavity, which is filled around it with gold foil in as careful a manner as any crown cavity, allowing the gold to overlap the margin so as to perfectly protect all of the root from the action of deleterious agents. By such means what in the case of ordinary wooden pivot would be the exposed part of the root is perfectly protected and inclosed by the gold filling, which at the same time gives support to the square cylinder in the centre of it. In placing the cylinder in the root with the pivot in it preparatory to inserting the gold filling about it in the cavity, the split or space between the two parts composing the pivot should range directly back, from the anterior to the posterior, and not from one approximal surface to the other. When this is done the pivot is drawn out from the cylinder which remains firmly fixed in the root, and that part of the cylinder, which may project, filed down to a level with the surface of the filling. An impression of this surface is then taken with wax or gutta-percha and a die and counter-die made of fusible metal by means of which a disk of platinum plate is swaged to fit accurately the concave surface of the gold filling in the root. When this is done, the convex surface of this disk is thinly covered with wax and the disk placed in its proper position over the gold filling in the root, and slightly pressed on it in order to obtain an impression by which to cut a square hole to correspond with the orifice of the square cylinder. After this square hole is cut in the disk, the outer end of the pivot is inserted in it, secured by means of wax, and the whole returned to the root (pivot in the cylinder) in order to make certain that the pivot is in its proper position, when it is carefully removed and secured by an investment of plaster and asbestos, in order that the pivot may be soldered to the disk.

This being done, the pivot and disk are again returned to the root and if found correct, the protruding part of the pivot above the concave surface of the disk is filed down to a level with this surface. This being done, the disk and pivot are returned to the cylinder in the root, and the plate tooth is placed in its proper position and attached to the disk by means of wax. The disk and pivot with the plate tooth thus attached are carefully removed from the root and invested in plaster and asbestos in order that a backing of gold may be made and the tooth thus soldered to it and the disk.

The tooth is now ready to be inserted, and by separating the two parts which form the pivot slighly at its apex or free extremity, this pivot will tightly fit the cylinder, the two halves acting as springs, which is the object in making the pivot of an alloy gold and platinum and also in two parts.

